# Tuberculosis of the Salivary Glands

**Published:** 1898-04

**Authors:** 


### Tuberculosis of the Salivary Glands.

Though it is easy to induce tuberculosis of the salivary glands experimentally, we meet the disease clinically but very seldom. O'Zoux describes two cases in the submaxillary gland (*Arch. clin. de Bordeaux*, No. 1, 1897). The swelling is considerably larger than in simple adenitis and is exceedingly painful; the pus is thin and moderate in amount. The author explains the rarity of the disease in this location by the fact that the food remains in contact with the oral mucous membrane for too short a time to give rise to infection.—*American Medico-Surgical Bulletin.*

